# A Phase 3 Study of Micafungin Versus Amphotericin B Deoxycholate in Infants With Invasive Candidiasis

**DOI:** 10.1097/INF.0000000000001996

**Published:** 2018-03-24

**Authors:** Daniel K. Benjamin, David A. Kaufman, William W. Hope, P. Brian Smith, Antonio Arrieta, Paolo Manzoni, Laura L. Kovanda, Christopher Lademacher, Brigit Isaacson, Deborah Jednachowski, Chunzhang Wu, Atsunori Kaibara, Thomas J. Walsh

**Affiliations:** From the * Department of Pediatrics, Duke University Medical Center, Durham, North Carolina; † Division of Pediatrics, University of Virginia Health System, Charlottesville, Virginia; ‡ Department of Molecular and Clinical Pharmacology, University of Liverpool, Liverpool, United Kingdom; § Department of Infectious Diseases, Children’s Hospital of Orange County, Orange County, California; ¶ Neonatology and NICU, Azienda Ospedaliera OIRM–Sant’Anna Hospital, Torino, Italy; ‖ Astellas Pharma Global Development, Inc., Northbrook, Illinois; ** Astellas Pharma Inc., Tokyo, Japan; †† Internal Medicine/Infectious Disease, Weill Cornell Medicine of Cornell University, New York, New York.

**Keywords:** amphotericin B deoxycholate, infants, invasive candidiasis, micafungin, neonates

## Abstract

**Background:**

Amphotericin B deoxycholate (AmB-D) is standard of care treatment for neonatal invasive candidiasis (IC). Micafungin (MCA) has broad-spectrum fungicidal activity against *Candida* spp. We compared the efficacy and safety of intravenous MCA with intravenous AmB-D and assessed the pharmacokinetics of MCA in infants >2–120 days of age with proven IC in a phase 3, randomized, double-blind, multicenter, parallel-group, noninferiority study (NCT00815516).

**Methods:**

Infants were randomized 2:1 to MCA (10 mg/kg/d) or AmB-D (1 mg/kg/d) for ≥21 days. Primary efficacy endpoint was fungal-free survival (FFS) 1 week after last study drug dose. MCA population pharmacokinetics included simulated area under the curve (AUC) at steady state and maximum plasma concentration after 2-hour infusion. AUC pharmacodynamic target exposure was 170 µg·h/mL.

**Results:**

Thirty infants received MCA (n = 20) or AmB-D (n = 10). The trial was terminated early because of slow recruitment. FFS was observed in 12 of 20 [60%; 95% confidence interval (CI): 36%–81%] MCA-group infants and in 7 of 10 (70%; 95% CI: 35%–93%) AmB-D-group infants. The most common treatment-emergent adverse events were anemia [MCA: n = 9 (45%); AmB-D: n = 3 (30%)] and thrombocytopenia [n = 2 (10%) and n = 3 (30%), respectively]. Model-derived mean AUC at steady state for MCA was 399.3 ± 163.9 µg·h/mL (95% prediction interval: 190.3–742.3 µg/mL); steady state and maximum plasma concentration after 2-hour infusion was 31.1 ± 10.5 µg/mL (95% prediction interval: 17.0–49.7 µg/mL). MCA exposures were above the AUC pharmacodynamic target exposure.

**Conclusions:**

Within the study limitations, infants with IC treated with MCA achieved similar FFS compared with AmB-D. Both agents were safe and well tolerated.

## Abstract 2

Supplemental Digital Content is available in the text.

Invasive candidiasis (IC) is a cause of significant morbidity and mortality in hospitalized infants^1–3^ and can result in severe neurodevelopmental impairment.^4^ Treatment-refractory IC can occur in infancy despite the use of conventional antifungal therapy with amphotericin B or fluconazole.^5^ There is a paucity of data on the efficacy, safety and pharmacokinetics (PK) of antifungal agents for the treatment of neonatal IC because of technical challenges and ethical issues. Direct extrapolation of data from studies in adults is not appropriate because of differences in PK and pharmacodynamics (PD). Nonetheless, several antifungal agents are used clinically, and agents recommended by infectious disease advisory boards for the treatment of neonatal IC include amphotericin B deoxycholate (AmB-D), liposomal amphotericin B, amphotericin B lipid complex, fluconazole and the echinocandins micafungin (MCA) and caspofungin.^6,7^ To date, no infectious disease expert bodies have recommended a specific antifungal agent for neonatal use; however, AmB-D, fluconazole and MCA are considered as the current therapeutic agents of choice for the treatment of IC in infants.^6,8,9^

AmB-D has been the standard of treatment for neonatal IC in the United States for many years,^7^ but nephrotoxicity is a dose-limiting toxicity.^10^ Amphotericin B formulations have not been optimized for use in infants, and limited available data suggest that AmB-D exhibits considerable PK variability among infants.^11,12^ The use of fluconazole in infants may be limited because of the resistance of some *Candida* spp., its frequent use as prophylaxis in extremely premature infants, its poor activity against fungal biofilms and the high doses required to reach therapeutic concentrations.^9,13–15^ In contrast, the echinocandins are broad-spectrum antifungal agents, of which MCA is the most studied in infants and is active against biofilms as well as fluconazole-resistant *Candida* spp.^16,17^ The recommended dose regimen in adults is 100 mg/d (approximately 2 mg/kg), which has a mean exposure of 100 µg·h/mL in adults, although an in vivo rabbit model of hematogenous *Candida* meningoencephalitis (HCME) suggested a higher AUC target of 170 µg·h/mL is necessary to adequately treat central nervous system (CNS) candidiasis.^18^ PK bridging studies conducted in infants ≤4 months of age^19^ suggested that the use of MCA at doses of 10 mg/kg/d is sufficient to reach the PD target in infants. PK and safety studies have demonstrated that MCA doses of up to 15 mg/kg/d are safe and well tolerated in young infants, offering exposure levels that are adequate to treat end-organ dissemination, including HCME.^18–20^ MCA is currently the only antifungal agent approved for neonatal use in the European Union,^21^ and in the United States, it is approved for the treatment of IC and candidemia in children ≥4 months old.^22^

Little is known about the comparative efficacy, safety and tolerability of MCA versus AmB-D in the treatment of neonatal IC. Herein, we report the efficacy and safety of MCA compared with AmB-D for the treatment of proven neonatal IC from a phase 3 study. A population PK analysis of MCA was also performed.

## MATERIALS AND METHODS

### Study Design and Patients

This was a phase 3, randomized, double-blind, multicenter, parallel-group, noninferiority study (ClinicalTrials.gov identifier: NCT00815516) comparing the efficacy and safety of intravenous MCA with intravenous AmB-D as treatment for IC in infants, and assessing the PK of intravenous MCA. Infants >2–120 days of life at the time of culture acquisition with proven IC were enrolled and randomized 2:1 to MCA or AmB-D. MCA was administered at 10 mg/kg/d, and AmB-D was dosed at 1 mg/kg/d for a minimum of 21 days,^23^ and for a maximum of 28 or 42 days in infants without or with end-organ dissemination, respectively. The dose rationale for MCA was based on data from previous preclinical and clinical studies.^18,20^ Each infant was assessed for 30 days after the last dose of study drug. The follow-up was performed to capture emergent and recurrent fungal infections, to obtain a final safety evaluation and to check for resolution of those IC episodes that had not resolved by the end of treatment. An end of study visit was performed 30 days after the final dose of study drug. An independent Data Review Panel reviewed and confirmed the diagnosis of IC and evaluated the efficacy outcome for each subject. Drug safety was evaluated by an unblinded Data Safety and Monitoring Board.

A diagnosis of IC required 1 of the following criteria to be met ≤4 days before the first dose of study drug: a positive blood culture collected for proven candidemia; a positive urine fungal culture by in and out catheterization (≥10^3^ colony-forming units/mL in a single urine culture) or suprapubic aspiration (≥10^2^ colony-forming units/mL in a single urine culture) for proven candiduria; the presence of *Candida* spp. in cerebrospinal fluid (CSF) for proven *Candida* meningitis; either a positive tissue biopsy or culture from a normally sterile body fluid (ie, blood, urine or CSF); or a positive culture for *Candida* (or yeast) obtained from a new drain (≤7 days before first dose of study drug) from a normally sterile site.

Exclusion criteria included any history of hypersensitivity or severe vasomotor reaction to any echinocandin or systemic amphotericin B product, receipt of >48 hours of systemic antifungal therapy before treatment for IC or breakthrough systemic fungal infection while receiving an amphotericin B product or an echinocandin as prophylaxis. In addition, infants who had previously failed systemic antifungal therapy for the current IC episode, including recurrence of the same *Candida* infection within 2 weeks of completing systemic antifungal treatment, were excluded, as were those who were coinfected with a non-*Candida* fungal organism or whose positive yeast cultures were solely from an indwelling bladder catheter or sputum, or with a concomitant medical condition that might create unacceptable additional risk in the opinion of the investigator and/or medical advisor.

The parent/legal guardian provided written informed consent. The study received approval from the Institutional Review Board/Independent Ethics Committee at each site and was conducted in accordance with the International Conference on Harmonisation Guidelines for Good Clinical Practice.

### Efficacy Assessments

The primary efficacy endpoint was fungal-free survival (FFS) 1 week after the last dose of the study drug, irrespective of treatment discontinuation. FFS was defined as an infant alive and fungal free with no requirement for continued treatment with alternative antifungal therapy. Secondary efficacy variables included clinical response of infants with clinical signs and symptoms of fungal infection at baseline and at 1 week after the last dose of study drug, mycologic response at the end of study drug therapy and at 1 week after the last dose of study drug and the frequency of emergent and recurrent fungal infections through the end of study.

Attributable signs related to fungal infection were assessed during screening, and clinical response was assessed weekly while on study drug therapy, at 1 week after the last dose of study drug and at the end of study visit. Responses were defined as follows: complete response (resolution of all attributable signs related to fungal infection); partial response (improvement in attributable signs related to the fungal infection); stabilization (minor improvement or no change in attributable signs related to the fungal infection and the infant continued on therapy without deterioration); progression (deterioration in attributable signs related to the fungal infection or death presumably related to fungal infection).

Mycologic response was assessed at the end of study drug therapy and 1 week after the last dose of study drug. Eradication was indicated by culture or the histologically documented absence of the infecting *Candida* spp. from all previously positive, normally sterile sites during therapy (2 negative samples collected ≤24 hours apart or 1 negative culture for *Candida* meningitis and/or candiduria, with no sign of fungal growth on culture ≤72 hours of collection). Persistence was defined by continued isolation or histologic documentation from a normally sterile site. Blood and urine fungal cultures were requested to be performed every 48 hours until 2 negative cultures were obtained, separated by ≥24 hours. When CSF culture was positive, repeat lumbar puncture was to be repeated every 4–7 days until negative culture was documented.

Following positive *Candida* cultures, end-organ dissemination was assessed using abdominal ultrasound and/or computed tomography scanning, echocardiogram, head imaging with ultrasound, magnetic resonance imaging or computed tomography scanning or ophthalmoscopy. An emergent fungal infection was defined as a non-*Candida* invasive fungal infection detected at any time during the study or any invasive fungal infection detected >96 hours postbaseline involving a *Candida* spp. other than those detected at baseline. Recurrent infection was defined as a systemic fungal infection with eradication on or before the end of study drug therapy but reappearing within 1 week later, in infants who developed positive blood cultures or a mycologically confirmed deep-seated *Candida* infection of the same species as that causing the infection at enrollment.

All fungal isolates were identified to the genus and species level using standard techniques. Briefly, each isolate was subcultured in CHROMagar *Candida* (Becton Dickinson and Company, Sparks, MD) and molecular identification by sequencing of the internal transcribed spacer region and/or 28S ribosomal subunit. In vitro susceptibility testing was performed on all fungal isolates collected during the study to calculate the minimum inhibitory concentrations (MICs) according to Clinical Laboratory Standards Institutes (CLSI) methodology (M27-A3) and European Committee on Antimicrobial Susceptibility Testing (EUCAST) methodology (EDef7.1).^24,25^

### PK Analysis

Up to 3 PK blood samples (≤300 µL) were collected from infants who participated in the PK portion of the study. A population PK approach was used to define the PK profile of MCA. A linear 2-compartment population PK model with body weight–based allometric scaling was developed in neonatal and pediatric patient populations by amending a previous model^19,26^ and including data from the current study in addition to 2 previous phase 1 studies and 1 previous phase 3 study in infants.^20,27,28^ Individual post hoc estimates of PK parameters, simulated area under the curve (AUC) at steady state and maximum plasma concentration at the end of 2-hour infusion at once-daily dose of MCA 10 mg/kg were derived based on the model. AUC PD target exposure was 170 µg·h/mL based on an in vivo rabbit model of HCME.^18^

### Safety Assessments

Adverse events, biochemical and hematologic laboratory parameters and survival were assessed for all infants who received at least 1 dose of study drug. Treatment-emergent adverse events (TEAEs), defined as AEs occurring from the first dose of the study drug through 72 hours after the last dose, were summarized by treatment group. Nephrotoxicity was potentially a key difference between the study drugs, which was defined as >25%, >50% and >100% change from baseline in serum creatinine.

### Statistical Analyses

Based on an estimated treatment success rate of 75%,^29^ a planned sample size of 225 infants was estimated to provide ≥90% power to demonstrate the noninferiority of MCA to AmB-D at a 2.5% significance level using a margin of 20%. For the primary efficacy analysis, which used the full analysis set (all randomized infants who were administered any amount of study drug), it was planned that a 2-sided 95% confidence interval (CI) would be constructed for the difference in success rates between MCA and AmB-D, based on the Cochran-Mantel-Haenszel method adjusting for estimated gestational age (<27 weeks, ≥27 weeks) and geographic region (North America/Europe, Latin America and other region). The safety analysis set included all randomized infants who received at least 1 dose of study drug. Demographic and baseline characteristics, secondary endpoints and safety endpoints were descriptively summarized by treatment group. Statistical analyses were performed using SAS version 9.1.3 (SAS Institute, Inc., Cary, NC).

## RESULTS

### Patient Demographics and Baseline Characteristics

The trial was conducted between February 23, 2013, and December 15, 2014, at 71 study centers in 23 countries. The trial was terminated before completion after 2 years and 5 months because of slow recruitment [including time from opening of first study center to screening (May 2012) until study termination (October 2014)]. However, 30 infants were randomized and received study drug (MCA: n = 20; AmB-D: n = 10). Patient demographics and baseline characteristics are summarized in Table, Supplemental Digital Content 1, http://links.lww.com/INF/D95. In both treatment groups, the most common comorbid conditions identified at baseline were respiratory distress, anemia and hyperbilirubinemia. The most common organisms were *Candida albicans* in 13 (41%) infants and *Candida parapsilosis* in 11 (34%) infants.

### Study Completion

Eleven (55%) MCA-treated infants and 4 (40%) AmB-D-treated infants completed the treatment period. The mean ± standard deviation treatment duration was 18.6 ± 10.7 days and 15.5 ± 11.2 days for the MCA and AmB-D treatment groups, respectively.

Overall, 9 (45%) infants discontinued treatment early with MCA and 6 (60%) infants discontinued treatment early with AmB-D. Reasons for early discontinuation of treatment included AEs [MCA: 3 (15%); AmB-D: 3 (30%)], physician decision [MCA: 3 (15%); AmB-D: 0], death [MCA: 2 (10%); AmB-D: 0], lack of efficacy [MCA: 1 (5%); AmB-D: 1 (10%)] or protocol violation [MCA: 0; AmB-D: 2 (20%)]. Physician decisions for discontinuing treatment with MCA included persistence of infection despite treatment (n = 1), protection of the subject’s best interests (n = 1) and a decision to switch treatment to another antifungal agent not used in the present study (n = 1). Sixteen (80%) MCA-treated infants and 9 (90%) AmB-D-treated infants completed follow-up. Four (20%) MCA-treated infants discontinued the study before the follow-up visit because of death in 3 (15%) infants, and physician decision based on the infant’s best interests in 1 (5%) infant. One (10%) AmB-D-treated infant discontinued the study because of death. Eleven infants received alternative systemic antifungal treatment after discontinuation of study treatment (MCA group, n = 4; AmB-D group, n = 7). In 2 of those infants (1 each in MCA and AmB-D groups), the alternative systemic antifungal treatments were initiated >48 hours after discontinuation of the study drug.

### Efficacy Outcomes

The primary efficacy outcome of FFS at 1 week after the last dose of study drug was observed in 12 (60%; 95% CI: 36%–81%) infants in the MCA group and in 7 (70%; 95% CI: 35%–93%) infants in the AmB-D group (Table 1). An additional 5 (25%) infants in the MCA group and 2 (20%) infants in the AmB-D group were alive at 1 week after the last dose of study drug, but were not fungal free and, therefore, did not meet the criteria for the primary efficacy end point. Of the 5 infants in the MCA group who were alive at 1 week after the last dose, 2 infants had at least 1 negative culture before the 1-week follow-up visit. One of 2 infants in the AmB-D group had 1 negative culture.

**TABLE 1.**
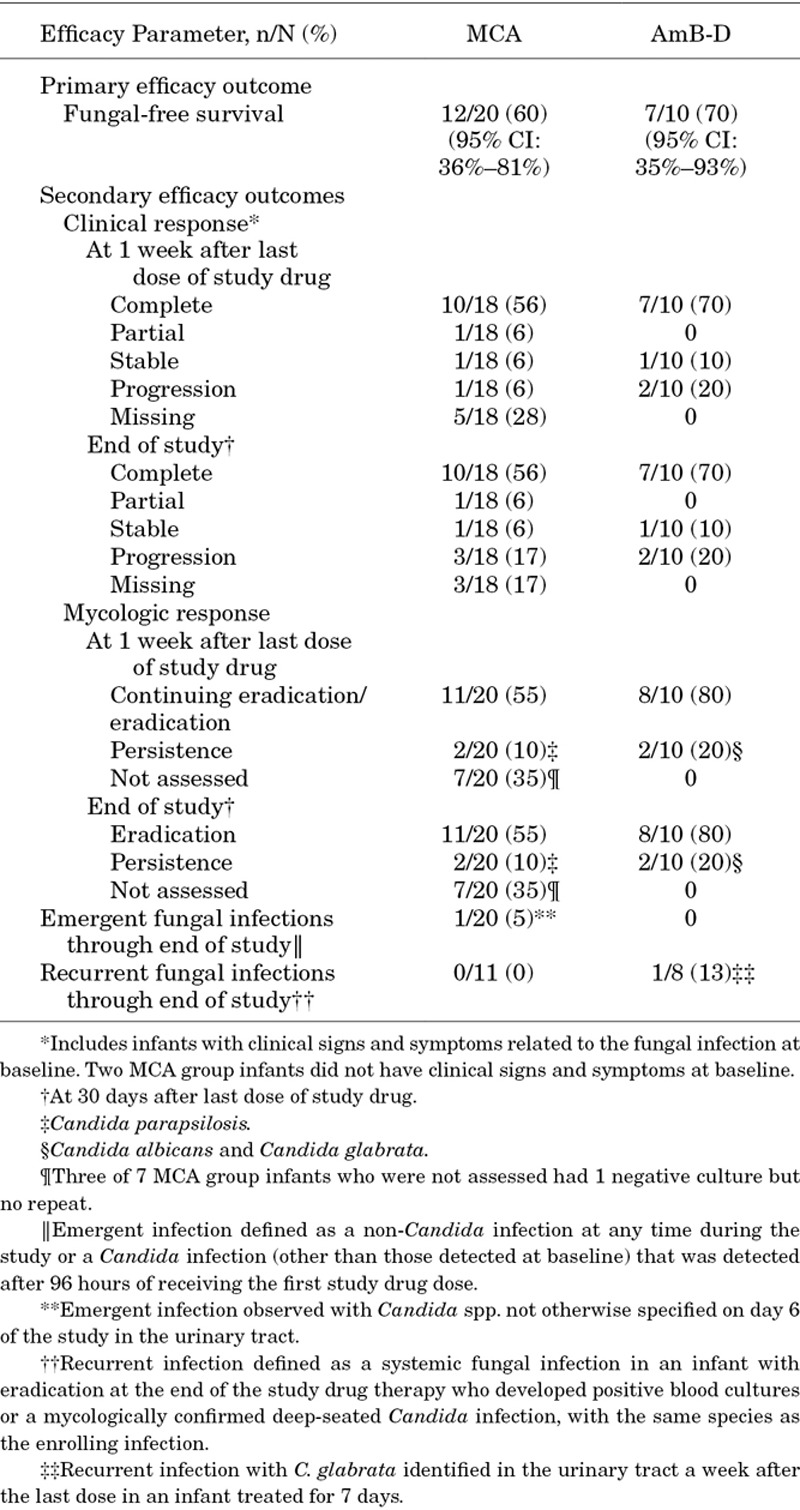
Efficacy Outcomes (Full Analysis Set)

Secondary efficacy outcomes are summarized in Table 1. Of the 18 infants in the MCA group and 10 infants in the AmB-D group with clinical signs and symptoms of fungal infection at baseline, 11 infants (61%; 95% CI: 36%–83%) compared with 7 infants (70%; 95% CI: 35%–93%), respectively, had a positive clinical response at the end of the study and 1 week after the last dose of study drug. Eleven (55%; 95% CI: 32%–77%) and 8 (80%; 95% CI: 44%–98%) of MCA- and AmB-D-treated infants, respectively, achieved eradication at the end of study drug therapy and 1 week after the last dose of study drug. One infant in the MCA group was deemed by the Data Review Panel as having met the primary efficacy outcome but had only 1 culture-negative sample collected during treatment and so eradication was not confirmed.

Persistent fungal infection occurred in 2 (10%) infants in the MCA and in 2 (20%) infants in the AmB-D groups, respectively. The persistent infections were because of *C. parapsilosis* in both MCA-treated infants and *Candida glabrata* and *C. albicans* in AmB-D-treated infants (Table 1). The MCA MICs of the baseline isolates for the 2 persistent infections in the MCA group were 1 mg/L (CLSI and EUCAST methodologies) for 1 infection and 1 mg/L (CLSI) and 0.5 mg/L (EUCAST) for the other infection (within the CLSI susceptible criteria for *C. parapsilosis* of ≤2 mg/L, and between the EUCAST susceptible and resistant breakpoints for *C. parapsilosis* of 0.002 and 2 mg/L, respectively). Both infants were enrolled with candidemia and received 19 and 16 days of study drug, respectively, before discontinuing treatment. The estimated duration of persistent fungal infection was 36 and 35 days, respectively. MICs for the follow-up isolates collected on day 19 for both MCA-treated infants did not increase (CLSI MIC value of 1 mg/L and EUCAST 1 and 0.25 mg/L). One of 2 MCA-treated infants was diagnosed on day 19 with end-organ dissemination to the CNS/brain based on head ultrasound, which revealed coagulation in ventricles and nodules consistent with fungal sepsis.

Similarly, infants in the AmB-D group with persistent infection caused by *C. glabrata* and *C. albicans* were treated for baseline candidemia with 11 and 10 days of study drug before discontinuing treatment. The estimated duration of persistent fungal infection was 21 and 19 days, respectively. The MIC values of both baseline isolates (1 mg/L CLSI methodology and 0.5 mg/L EUCAST methodology) were at the CLSI and EUCAST susceptibility breakpoint of 1 mg/L. The infant with *C. glabrata* had subsequent isolates with identical MICs to baseline. This infant also had end-organ dissemination to the peritoneal cavity diagnosed on day 15. Similarly, the infant with *C. albicans* candidemia had follow-up cultures on day 9 but MICs had not changed from baseline (1 mg/L CLSI method and 0.25 and 0.5 mg/L for EUCAST methodology). This infant was also diagnosed on day 11 with end-organ dissemination in the bowel.

Five (17%) infants had isolated candiduria at enrollment [MCA, n = 3 (15%); AmB-D, n = 2 (20%)]. All infants responded to treatment except 1 MCA-treated infant, who had 1 positive urine culture (negative blood culture) 3 days before starting MCA. Ten MCA doses were administered before discontinuation per physician decision. No urine cultures were reported after starting MCA and so the response was not assessable (ie, ruled a failure). Both of the other MCA-treated infants had no growth on follow-up urine cultures.

Emergent fungal infection was observed in 1 (5%) MCA-treated infant during study treatment. This involved an infection of the urinary tract by a *Candida* spp. The isolate was not sent to the central laboratory; therefore, identification of the species could not be confirmed. No recurrent infections were observed in MCA-treated infants throughout the study; however, recurrent infection with the same species (*C. glabrata*) was noted in 1 (10%) AmB-D-treated infant which was present 1 week after the end of treatment with a localized urinary tract infection (UTI). FFS in infants with baseline end-organ dissemination was achieved in 3 of 7 (43%; 95% CI: 10%–82%) MCA-treated infants and 1 of 3 (33%; 95% CI: 1%–91%) AmB-D-treated infants, both at the end of study drug therapy and 1 week after the last dose of study drug.

### Pharmacokinetics

A population PK model was developed using data from this and 8 other studies (data on file at Astellas). A 2-compartment model with body weight–based allometric scaling and first-order elimination adequately described MCA PK data regardless of age. In this study, PK data were collected from 12 of 30 study participants (Table 2). The mean AUC at steady state and maximum plasma concentration after 2-hour infusion of MCA were 399.3 ± 163.9 µg·h/mL (95% prediction interval: 190.3–742.3 µg/mL) and 31.1 ± 10.5 µg/mL (95% prediction interval: 17.0–49.7 µg/mL), respectively, for the 12 participants in this study. All exposures after MCA 10 mg/kg/d were above the AUC PD target exposure of 170 µg·h/mL.

**TABLE 2.**
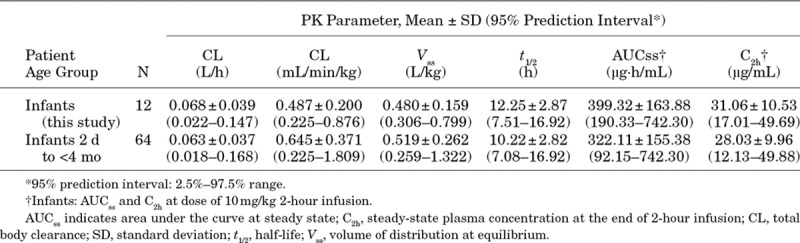
Summary of Individual Pharmacokinetic Parameters and Exposure Parameters of MCA 10 mg/kg

### Safety Outcomes

Overall, 18 (90%) and 9 (90%) of MCA- and AmB-D-treated infants, respectively, experienced ≥1 TEAE, with 11 (55%) MCA-treated infants and 5 (50%) AmB-D-treated infants experiencing a TEAE considered by investigator assessment to be possibly or probably related to study drug (Table 3). The most common TEAEs (irrespective of relatedness to study drug) were anemia [MCA: n = 9 (45%); AmB-D: n = 3 (30%)] and thrombocytopenia [MCA: n = 2 (10%); AmB-D: n = 3 (30%)]. In all MCA-treated infants and 1 of 3 AMB-D-treated infants thrombocytopenia was resolved. Of the 2 AMB-D-treated infants in whom thrombocytopenia was not resolved, 1 was not fungal free at 1 week after the end of therapy. The frequency, nature and severity of individual TEAEs were mostly similar between groups. At least 1 serious AE (SAE) occurred in 12 (60%) and 7 (70%) MCA-treated and AmB-D-treated infants, respectively, with anemia being the most common SAE in the MCA group [n = 4 (20%)]. All cases of anemia resolved. Three (15%) MCA-treated infants and 1 (10%) AmB-D-treated infant died during the study. Deaths in the MCA group were attributed to gastrointestinal necrosis, cardiovascular insufficiency and septic shock after sepsis reported at baseline [n = 1 for each, estimated gestation age (EGA) at birth: 22, 26 and 28 weeks, respectively). One death in the AmB-D group (EGA at birth: 24 weeks) was attributed to necrotizing enterocolitis. The investigators reported that none of the deaths in either group were considered to be related to the study drug.

**TABLE 3.**
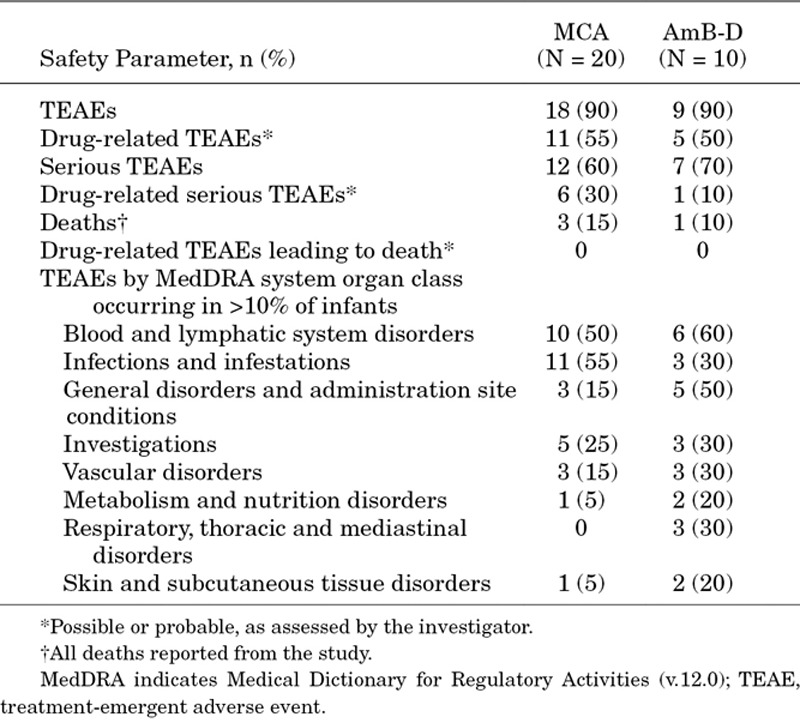
Summary of Adverse Events (Safety Analysis Set)

Changes in hematology and biochemistry parameters from baseline are shown in Table 4. Bilirubin and alanine transaminase levels were elevated in a greater proportion of the MCA group. Electrolyte supplementation was received by 6 (30%) MCA-treated infants and by 5 (50%) AmB-D-treated infants [potassium supplementation: n = 2 (10%) and n = 3 (30%) and magnesium supplementation: n = 3 (15%) and n = 2 (20%), respectively].

**TABLE 4.**
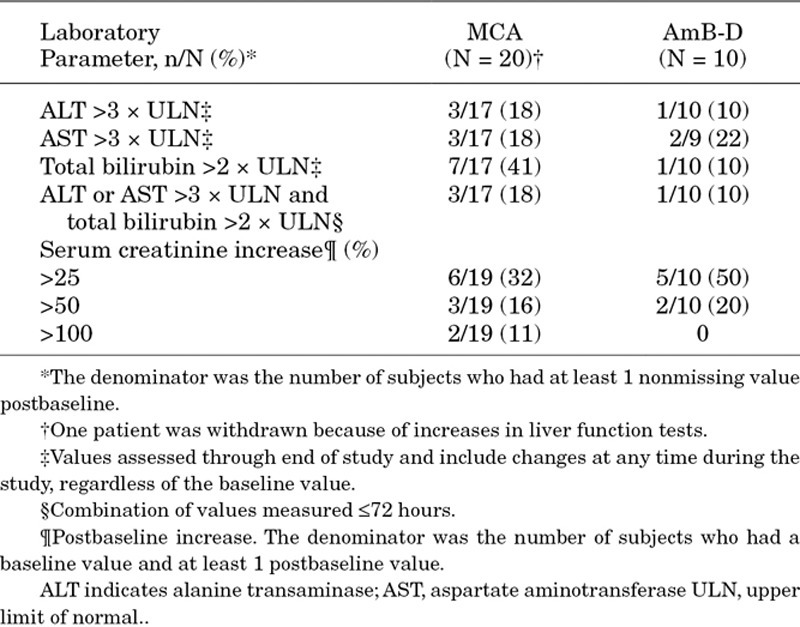
Summary of Clinical Laboratory Test Findings (Safety Analysis Set)

## DISCUSSION

Despite the study being terminated early because of poor recruitment, the key finding was that more than half of infants with IC including candidemia responded to treatment with MCA or AmB-D (60% vs. 70%, respectively), as assessed by the primary endpoint of FFS at 1 week after the last dose of study drug. Consistent with the small sample size in each treatment arm, the 95% CIs of the point estimates of infants in each group who responded to treatment were wide but overlapped. The proportions of infants with a successful clinical response after MCA or AmB-D (61% vs. 70%, respectively) were similar to the primary endpoint of FFS 1 week after last dose of study drug. Although the low sample number makes interpretation of the data difficult, efficacy in the treatment of IC and candidemia was observed in both the MCA and AmB-D groups, regardless of fungal species, infection site, age, gender, race, gestational age or geographic region of the infant. A smaller percentage of MCA-treated infants achieved a mycologic response at the end of study drug therapy and 1 week after the last dose of study drug compared with AmB-D infants. However, mycologic failure in 7 of the 9 MCA-treated infants was attributable to a lack of assessment of mycologic response, whereas, in all AmB-D-treated infants, mycologic failure was indicative of persistent fungal infection. All 7 MCA-treated infants not assessed for mycologic response were alive at 1 week after the last dose, with 3 of the 7 having at least 1 negative culture (the study required 2 negative cultures for confirmation of eradication).

Persistent *Candida* infections caused by *C. parapsilosis* in 2 MCA-treated infants and *C. glabrata* and *C. albicans* in AmB-D-treated infants (n = 1 for each) were documented. *C. parapsilosis* is responsible for 1 quarter of invasive fungal infection cases in very low birthweight infants (<1500 g),^30^ and has emerged as the most common non-*albicans Candida* species in neonatal IC.^31^ Though the suitability of the echinocandins for clinical management of *C. parapsilosis* is uncertain because of higher MICs compared with other *Candida* species,^7^ there was no discernible difference in the frequency and species of persistent fungal infections in this study.

High rates of survival and successful treatment responses to MCA among patients <18 years of age with IC have been reported in prospective, observational studies.^32,33^ Success rates in pediatric studies are similar to the findings of our study in infants. For example, in a pediatric substudy from a randomized, double-blind study comparing MCA (2 mg/kg) with liposomal amphotericin B (3 mg/kg) in patients <16 years of age with IC, the investigators reported similar treatment success rates after MCA (defined as both a clinical and mycologic response at the end of therapy; 73% compared with 76% for liposomal amphotericin B).^34^

The population PK model that was developed to define the PK of MCA showed that the weight-adjusted total body clearance was higher in infants than in older pediatric patients, possibly because of the fact that total body clearance per individual increased less than proportionally to the weight increase.^18,20,35^ MCA at dosages of as much as 10 mg/kg demonstrate linear PK and favorable tolerability in infants (<4 months of age), while also providing exposure levels adequate for CNS coverage.^18–20,27,35,36^ This is supported by the fact that the systemic plasma exposure at 10 mg/kg in infants was predicted to exceed that shown to result in antifungal activity in the cerebrum and cerebellum of a rabbit model (AUC_0–24_, 170 µg·h/mL).

Previous studies using MCA 10 mg/kg IV in the neonatal population demonstrated that this dosage confers plasma exposure to safely treat HCME.^19,20^ The range of weights of infants with candidemia within this study were well within the population that can safely receive the 10 mg/kg dosage of MCA.

Generally, MCA and AmB-D demonstrated acceptable tolerability, with no notable differences between groups reported when individual AEs and SAEs were compared. There was a slightly greater degree of nephrotoxicity at >25% baseline caused by AmB-D, although AmB-D-induced nephrotoxicity, defined as elevated serum creatinine, in the infant is usually reversible.^37^ In understanding the relative tolerability of AmB-D in infants within the neonatal intensive care unit compared with older children, there are several hypotheses, including a greater nephronal reserve or the attention to appropriate hydration in the intensive care unit.^38^ While nephrotoxicity may be more limited in infants compared with that observed in older children, tubular toxicity is a problem associated with AmB-D in both populations.^39^ Evidence also exists to support the safety of MCA up to 10 mg/kg in the pediatric patient population as, for example, a systematic review of 9 clinical trials in premature and nonpremature infants <2 years of age found that treatment-related TEAEs were reported in only 23% of patients and did not differ between premature and nonpremature infants.^40^ Although the rate of TEAEs and treatment-related TEAEs in the MCA arm of this study were higher than those historic data, interpretation is limited by the low enrolment in this study, and may reflect in part differences in treatment dose and duration compared with those studies.

*Candida* UTIs are commonly present in neonatal candidemia. Preclinical models and clinical observations demonstrate that echinocandins are able to eradicate *Candida* spp. causing renal candidiasis, reflecting their relatively high concentrations in renal parenchyma.^41–43^ Although only a small fraction of a circulating echinocandin is typically excreted in the urine, sufficiently high urinary concentrations above the MIC of most isolates are achieved to successfully treat *Candida* lower UTIs.^44,45^

A major limitation of the study was, of the planned recruitment of 225 infants, only 30 infants were enrolled and received the study drug, thereby preventing inferential statistical comparisons to establish the noninferiority of MCA compared with AmB-D for the treatment of neonatal IC. Therefore, interpretation of the results presented is based on a descriptive summary of the findings according to treatment group.

Recruitment was substantially impacted by the low incidence of fungal infection in this patient population. This was because of the use of preventive measures associated with a decreasing incidence of IC in neonatal intensive care units, including fluconazole or nystatin prophylaxis, and improvements in infection control that have—more generally—reduced nosocomial infections in neonatal intensive care unit patients.^46^

Furthermore, several other factors contributed to the low patient enrollment. Seventy-one of 597 (12%) global investigational sites contacted by the Sponsor agreed to participate. Of the 71 planned sites, 66 sites were not able to enroll any infants. Recruitment of the small target population was further impacted by local ethics committee and/or country-specific restrictions relating to conducting pediatric studies at the institution, the comparator used and the safety profile of the comparator, the product label for both MCA and comparator and the risks associated with each compound. The use of AmB-D in place of liposomal amphotericin B or fluconazole was frequently denied per local clinical practice, possibly because of perceptions of improved safety despite the adverse effects of AmB-D, nephrotoxicity in particular, being less than those in older children and adults.^47^ In addition, the efficacy and safety of AmB-D are not well established in this patient population, and the MCA dose (10 mg/kg) proposed in this study is higher than the dose approved at the time of the study in Europe (2–4 mg/kg) for pediatric patients including infants, with limited safety data available for this dosage level. The approved MCA dose in Europe has subsequently been amended to include higher dosages to treat IC (4–10 mg/kg) in infants <4 months of age. Results for this study show that MCA at a daily dose of 10 mg/kg was well tolerated. This finding is consistent with other published safety findings of MCA in infants.^20^

In conclusion, this study found that infants with IC including candidemia who were treated with MCA achieved numerically similar FFS and clinical response to those infants treated with AmB-D. Both agents appeared to be safe and well tolerated. These results must be interpreted with caution because of low enrollment into the study and the subsequent loss of statistical power required for inferential statistical testing.

## Supplementary Material
