# Supplementary material for: A Phase 3 Study of Micafungin Versus Amphotericin B Deoxycholate in Infants With Invasive Candidiasis
Source: Pediatr Infect Dis J. 2018 Mar 24;37(10):992–8. doi: 10.1097/INF.0000000000001996 (PMC6155365; doi:10.1097/INF.0000000000001996)
Supplement: Supplementary file 1 [file inf-37-992-s001.docx]

**Supplemental Digital Content 1.** Patient demographics and baseline characteristics (full analysis set)

| **Parameter** | **MCA (N = 20)** | **AmB-D (N = 10)** |
| --- | --- | --- |
| Male, n (%) | 8 (40) | 6 (60) |
| Race, n (%) |  |  |
| White | 18 (90) | 9 (90) |
| Other* | 2 (10) | 1 (10) |
| Age, days, median (range) | 18 (9–117) | 16 (12–26) |
| Age group, n (%) |  |  |
| ≤4 weeks | 15 (75) | 10 (100) |
| >4 weeks to 4 months | 5 (25) | 0 |
| Gestational age, n (%) |  |  |
| <27 weeks | 3 (15) | 2 (20) |
| ≥27 weeks | 17 (85) | 8 (80) |
| Region, n (%) |  |  |
| North America/Europe | 15 (75) | 9 (90) |
| Latin America/Mexico | 4 (20) | 1 (10) |
| Other | 1 (5) | 0 |
| Birth weight, g, mean ± SD | 1807 ± 879 | 2171 ± 1009 |
| Risk factors, n (%) |  |  |
| Assisted mechanical ventilation | 18 (90) | 7 (70) |
| Enteral feeding | 16 (80) | 7 (70) |
| Indwelling vascular catheters | 15 (75) | 9 (90) |
| Carbapenem or 3^rd^ generation cephalosporin ≤7 days prior to positive fungal culture | 15 (75) | 5 (50) |
| Total parenteral feeding | 13 (65) | 9 (90) |
| Type of fungal infection, n (%) |  |  |
| Candidemia^†^ | 12 (60) | 7 (70) |
| IC^‡^ | 8 (40) | 2 (20)^§^ |
| Site of fungal infection, n (%) |  |  |
| Blood | 16 (80) | 7 (70) |
| Eye | 1 (5) | 0 |
| Urinary tract | 7 (35) | 2 (20) |
| Other | 1 (5) | 0 |
| *Candida* spp.^¶^, n (%) |  |  |
| *Candida albicans* | 8 (40) | 5 (50) |
| *Candida parapsilosis* | 9 (45) | 2 (20) |
| *Candida tropicalis* | 1 (5) | 1 (10) |
| *Candida glabrata* | 0 | 2 (20) |
| Other *Candida* spp.*^ǁ^* | 3 (15) | 1 (10) |
| DRP-confirmed presence of end-organ dissemination, n (%) |  |  |
| Yes | 7 (35) | 3 (30) |
| CNS/Brain | 2 (10) | 0 |
| Eye | 1 (5) | 0 |
| Kidney | 1 (5) | 0 |
| Liver | 1 (5) | 0 |
| Meninges/CSF | 0 | 1 (10) |
| Other | 4 (20) | 2 (20) |

*Includes Black/African American, Asian, and Middle-Eastern.

^†^Defined as *Candida* infection isolated from blood only.

^‡^Defined as *Candida* infection not solely isolated from blood.

^§^Information missing on 1 infant.

^¶^An infant could have infection caused by >1 *Candida* spp.

*^ǁ^*Includes *Candida famata*, *Candida guillermondii*, and *Candida lusitaniae*.

AmB-D indicates amphotericin B deoxycholate; CNS, central nervous system; CSF, cerebrospinal fluid; DRP, Data Review Panel; IC, invasive candidiasis; MCA, micafungin; SD, standard deviation.
